# Isolation, Characterization and Genomic Analysis of a Novel Jumbo Phage, AerS_266, That Infects *Aeromonas salmonicida*

**DOI:** 10.3390/microorganisms11112649

**Published:** 2023-10-28

**Authors:** Vera Morozova, Igor Babkin, Yuliya Kozlova, Artem Tikunov, Tatiana Ushakova, Alevtina Bardasheva, Valeria Fedorets, Elena Zhirakovskaya, Nina Tikunova

**Affiliations:** 1 Institute of Chemical Biology and Fundamental Medicine Siberian Branch of Russian Academy of Sciences, Novosibirsk 630090, Russia; morozova@niboch.nsc.ru (V.M.); arttik@ngs.ru (A.T.);; 2 Faculty of Natural Sciences, Novosibirsk State University, Novosibirsk 630090, Russia

**Keywords:** *Aeromonas salmonicida*, *Aeromonas* phage, phiKZ-like, chimallin, PhuZ, multisubunit RNA polymerase

## Abstract

*Aeromonas salmonicida* is the causative agent of septicemia in fish, and it is associated with significant economic losses in the aquaculture industry. While piscine *Aeromonas* infections are mainly treated with antibiotics, the emergence of resistance in bacterial populations requires the development of alternative methods of treatment. The use of phages can be one of them. A novel *A. salmonicida* jumbo phage, AerS_266, was isolated and characterized. This phage infects only mesophilic *A. salmonicida* strains and demonstrates a slow lytic life cycle. Its genome contains 243,674 bp and 253 putative genes: 84 encode proteins with predicted functions, and 3 correspond to tRNAs. Genes encoding two multisubunit RNA polymerases, chimallin and PhuZ, were identified, and AerS_266 was thus defined as a phiKZ-like phage. While similar phages with genomes >200 kb specific to *Aeromonas hydrophila* and *Aeromonas veronii* have been previously described, AerS_266 is the first phiKZ-like phage found to infect *A. salmonicida*.

## 1. Introduction

The genus *Aeromonas* belongs to the family *Aeromonadaceae* (order *Aeromonadales*). This genus is the prototype genus of the family, and it currently comprises 32 species (https://lpsn.dsmz.de/genus/aeromonas; accessed on 15 September 2023). *Aeromonas* spp. are widely distributed in aquatic ecosystems. In addition, these bacteria can be isolated from a variety of other sources such as seafood, meat, dairy products, vegetables, chlorinated water, and hospital water supplies [1]. Moreover, some aeromonads can survive and grow in food products when cooled in various packaging atmospheres in a wide range of pH and preservatives; therefore, the *Aeromonas* spp. has held the title “emerging foodborne pathogen”. These bacteria are able to produce biologically active extracellular enzymes and toxins; in addition, they are capable of biofilm formation. At least 19 members of this genus have been recognized as pathogens causing a wide range of infections in humans and animals [1,2]. Almost all members of the genus *Aeromonas* are mesophilic (grow at temperatures of 25 °C and above), with the exception of four *A. salmonicida* subspecies, namely *salmonicida*, *masoucida*, *smithia*, and *achromogenes*, whose growth is usually restricted to temperatures below 25 °C [3].

Bacteria belonging to the genus *Aeromonas* include numerous fish pathogens, the most important of which are *A. salmonicida* and *A. hydrophila* isolates, as they are responsible for severe infections in a wide range of fish species [1]. *A. salmonicida* is the causative agent of septicemia in fish, and this agent is associated with considerable economic losses in the aquaculture industry worldwide [4]. *Aeromonas* infections in aquaculture are now treated with antibiotics and antimicrobial chemicals; however, the emergence of resistance in bacterial populations requires the development of alternative treatments [5]. Phage therapy can replace or supplement antibiotic therapy in aquaculture. Published studies of phage treatment of *Aeromonas* infections in fish were based on bacteriophages that target *A. hydrophila* and *A. salmonicida* subsp. *salmonicida* [6,7,8]. However, aquatic pathogens are diverse and often include a mixture of *Aeromonas* species [9,10]. Therefore, it is essential to expand a search for new phages specific to bacteria of the *Aeromonas* genus. 

More than a hundred *Aeromonas* phages have been isolated, and most of their complete genomes are available in the GenBank database (https://www.ncbi.nlm.nih.gov/genomes/GenomesGroup.cgi?taxid=10239&host=bacteria, accessed 15 August 2023). Among them, several so-called giant *Aeromonas* phages have been discovered and studied [11,12,13,14,15,16,17,18].

Giant (jumbo) phages with lengths of >200 kb have been grouped into the heterogeneous cluster. This cluster contains phages of both myovirus and siphovirus morphotypes and is divided into three groups depending on their genetic composition. The genomes of the phages from different groups exhibit no similarity, which suggests the independent evolution of each group [19]. Group 1 includes the classic jumbo phages, and their prototype is the *Pseudomonas aeruginosa* phage phiKZ [20]. Characteristic proteins of this group are the multisubunit “double-barrel” RNA polymerase (RNAP), an unusual DNA polymerase from a family B DNA polymerases, DnaB-helicase, and phiKZ-like major capsid protein and terminase. Most phages from this group infect gammaproteobacteria. Group 2 is characterized by the classic family B DNA polymerase, gp23-type major capsid protein, and the presence of phage-encoded sigma factors instead of the multisubunit RNAP. Group 2 is divided into two subgroups: Subgroup 2.1 mainly includes phages specific to cyanobacteria, whereas Subgroup 2.2 contains phages that target gammaproteobacteria. Group 3 is also separated into two subgroups, with subgroup 3.1 identified by the presence of the T7-type DNA polymerase, whereas DNA polymerase III, similar to the bacterial replicative enzyme, is a type feature for subgroup 3.2 [19]. Group 1 and Group 2 contain phages with myovirus morphology; Group 3 includes myoviruses and siphoviruses. 

All known giant *Aeromonas* phages are myoviruses and belong to Groups 1 and 2. Group 1, the so-called phiKZ-like group, includes 10 *Aeromonas* phages. Nine of these infect *A. hydrophila*, and one, the AVP phage, uses *Aeromonas veronii* as a host. No phiKZ-like *A. salmonicida* phages were found. In contrast, the second group contains six phages that infect *A. hydrophila*, seven *A. salmonicida*-specific phages, and one more phage, *Aeromonas* phage AHP-1, which infects both *A. hydrophila* and *A. salmonicida* strains [17]. Group 2 phages are members of the *Straboviridae* family according to the latest version of the ICTV taxonomy release [21]. They are related to the coliphage T4 and possess no similarity with the Group 1 *Aeromonas* phages [19].

Here, we describe for the first time a novel phiKZ-like phage, AerS_266, and its host strain *A. salmonicida* CEMTC 4537, both of which were isolated from a polluted pond in Novosibirsk, Russian Federation.

## 2. Materials and Methods

### 2.1. Bacteria and Phage Isolation

Both the phage and its bacterial host were isolated from the same water sample, taken from a polluted pond in Novosibirsk, Russian Federation. Tenfold dilutions of water sample were prepared in sterile phosphate-buffered saline (pH 7.5), spread on Nutrient agar plates (Condalab, Madrid, Spain), and incubated for 24 h at 25 °C. Individual bacterial colonies were passaged three times under the same conditions. The bacterial species was identified via sequencing 1308 bp fragment of the 16S rRNA gene using primers 8F 5′-AGRGTTTGATCCTGGCTCA-3′ and 1350R 5′-GACGGGCGGTGTGTACAAG-3′ as described previously [22]. The obtained strain was deposited in the Collection of Extremophilic Microorganisms and Type Cultures (CEMTC) of the ICBFM SB RAS as *Aeromonas salmonicida* CEMTC 4537. Afterward, the water sample was sterilized via filtration through a 0.22 µM filter (Millipore, Guyancourt, USA), and the filtrate was screened for bacteriophages by spotting 10 µL aliquots onto a fresh layer of *A. salmonicida* CEMTC 4537 in the top agar. The plates were incubated overnight at 25 °C, and each plaque was suspended in sterile PBS to extract phage particles. Tenfold dilutions of obtained phage suspensions were spotted onto the fresh layer of *A. salmonicida* CEMTC 4537 to obtain single-phage plaques for subsequent phage extraction. The cycle of phage dilution and extraction was repeated three times.

### 2.2. Phage Propagation

*A. salmonicida* CEMTC 4537 was grown in Nutrient Broth (Condalab, Madrid, Spain) at 25 °C with shaking. The phage AerS_266 was added to the exponentially growing culture of CEMTC 4537 (OD_600_ = 0.4) with a multiplicity of infection (MOI, i.e., the ratio of phage to bacterium) of 0.1. The infected culture was incubated at 25 °C for 30 min without shaking and then with shaking until cell lysis appeared. Afterward, bacterial debris and cells were pelleted via centrifugation, and phage particles were concentrated from a supernatant using polyethylene glycol 6000 (PEG 6000; AppliChem, Darmstadt, Germany) precipitation as described previously [23].

### 2.3. Phage Plaques and Phage Particle Morphology 

The morphology of the AerS_266 plaques was determined in the top agar using the host culture *A. salmonicida* CEMTC 4537 after overnight incubation at 25 °C. In order to examine the phage AerS_266 particles’ morphology, a negative staining electron microscopy technique was applied as described previously [24]. The phage suspension (10^9^ pfu/mL) was adsorbed on a copper grid and contrasted using 1% uranyl acetate; afterward, the grid was examined for phage particles with a JEM 1400 transmission electron microscope (JEOL, Tokyo, Japan).

### 2.4. Biological Properties and Host Range Assay 

All experiments on the biological properties of the phage AerS_266 were performed twice, three times in each repeat. The statistical analysis and graphs were prepared using GraphPad Prizm v. 8.0 (https://www.graphpad.com (accessed on 31 August 2023). The investigation of biological properties and host range was performed as described previously with slight modifications, including incubation of most *Aeromonas* spp. cultures at 25 °C [25,26]. In brief, to perform burst size experiments, phage particles were added to the exponentially growing cell culture of the bacterial host *A. salmonicida* CEMTC 4537 with a MOI of 0.001 pfu per cell. The mixture was incubated for 5 min at 25 °C for phage adsorption, and afterward, bacterial cells were pelleted via centrifugation and resuspended in 10 mL Nutrient Broth (Condalab, Spain). The infected culture was incubated with shaking for 1 h at 25 °C. Culture aliquots were collected every 5 min, and the phage AerS_266 titer was determined. The lytic activity assay of the phage AerS_266 was carried out using the exponentially growing culture of *A. salmonicida* CEMTC 4537 that was mixed with the phage AerS_266 (0.01 pfu per cell). The mixture was then incubated with shaking at 25 °C. Aliquots were taken every 30 min, and the appropriate dilutions were spread on agar plates and incubated for 24 h at 25 °C. The next day, bacterial colonies were counted. Using the data obtained, a multistep bacterial killing curve for the phage AerS_266 was calculated. A spot-assay method [27] was applied to determine the host range for the phage AerS_266, and strains of *Aeromonas* spp. from the CEMTC of the ICBFM SB RAS (Novosibirsk, Russia) were investigated.

### 2.5. AerS_266 Complete Genome Sequencing and Analysis

The AerS_266 DNA was extracted from the phage preparation as described previously [28]. Briefly, phage particles were pelleted via centrifugation; then, the pellet was dissolved in an STM buffer containing 10 mM of NaCl, 50 mM of Tris-HCl, and 1 mM of MgCl_2_ (pH 8.0). Furthermore, the phage preparation was incubated with 5 mkg/mL of both RNase and DNase (Thermo Fisher Scientific, Waltham, MA, USA) for 1 h at 37 °C. Next, SDS, proteinase K (Thermo Fisher Scientific, Waltham, MA, USA), and EDTA were added to final concentrations of 0.5%, 100–200 mkg/mL, and 20 mM, respectively, and the mixture was incubated for 3 h at 55 °C. Finally, phenol–chloroform DNA extraction was carried out, and DNA was purified using ethanol precipitation. A paired-end genome library was obtained using a Nextera DNA Sample Preparation Kit (Illumina, Inc., San Diego, CA, USA), and sequencing was performed using the MiSeq Benchtop Sequencer and MiSeq Reagent Kit v.1 (Illumina Inc., USA). The genome was assembled de novo using the SPAdes Genome Assembler v.3.15.2 (http://cab.spbu.ru/software/spades, accessed on 18 February 2023). Rapid Annotation Subsystem Technology (RAST) v.2.0 (https://rast.nmpdr.org, accessed on 5 June 23) was used to annotate the putative genes. The obtained genes were verified manually using BLAST against sequences, deposited in the NCBI GenBank database. In addition, InterProScan [29], HHPred, and HMMER tools [30] were applied for the identification of hypothetical proteins. Comparative genome analysis was carried out using ViPTree (https://www.genome.jp/viptree, accessed on 10 August 2023) and VIRIDIC tools (http://rhea.icbm.uni-oldenburg.de/VIRIDIC, accessed on 15 August 2023). PhageTerm v.1.0.12 was applied to determine the position of the phage termini [31]. Search for virulence factors and antibiotic resistance genes was carried out using the Virulence Factor (VR) Database, (http://www.mgc.ac.cn/VFs, access date 31 August 2023), and the Antibiotic Resistance Gene (AGR) Database (https://card.mcmaster.ca/rgi, access date 30 August 2023), respectively. Dot-plot analysis of the protein sequences was performed using the MAFFT tool (https://mafft.cbrc.jp/alignment/server/index.html, access date 10 September 2023). The phylogenetic analysis of the essential proteins encoded by the AerS_266 genome was carried out as follows: the most similar protein sequences identified with BLASTP search were extracted from the NCBI GenBank database, and then the sequences were aligned and analyzed using MEGA 11.0 [32].

## 3. Results 

### 3.1. The Phage AerS_266 Biological Properties, Host Range Assay, and Phage Particle Morphology

**Figure 1:**
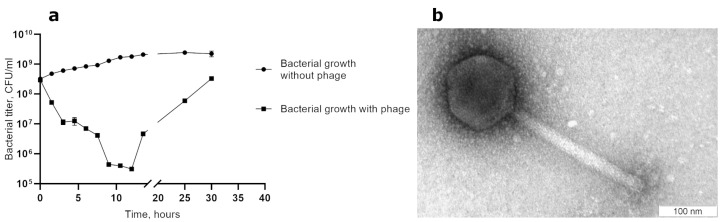
Biological properties and capsid morphology of the phage AerS_266: (**a**) the multistep bacterial killing curve for the phage AerS_266, obtained using its host strain *A. salmonicida* CEMTC 4537; (**b**) electron micrograph of the AerS_266 phage particles negatively stained with 1% uranyl acetate.

The AerS_266 phage formed clear small plaques on the layer of the top agar containing the host culture *A. salmonicida* CEMTC 4537. This phage infected 4 mesophilic *A. salmonicida* strains out of the 13 *A. salmonicida* and 32 other tested *Aeromonas* spp. (the list of the *Aeromonas* strains is available in Supplementary Table S1). A one-step growth experiment revealed a latent period of 30 min with a burst size of ~35 phage particles per infected cell. A multistep bacterial killing curve for the phage AerS_266 showed that the number of living bacteria decreased by three orders nine hours after infection and then increased (Figure 1a). The obtained results indicated that AerS_266 is a virulent phage with a slow lytic life cycle.

Electron microscopy revealed that the AerS_266 particle consists of a large capsid (Ø125 nm) connected to a long contractile tail (L = 270 nm). Therefore, the virion morphology corresponds to the myovirus morphotype (Figure 1b).

### 3.2. Genome Characteristics

The AerS_266 phage genome was sequenced and assembled de novo using the SPAdes Genome Assembler v.3.15.2. As a result of the assembly, the contig with an average coverage of 102 was obtained. The length of the AerS_266 genome was 243,674 bp; the GC content was calculated as 36.8% in contrast to the GC content of the host, *A. salmonicida*, which was found to be 58.48% [33]. This fact suggests that the AerS_266 molecular machine is mainly independent of the host cell. Phage term analysis revealed that the AerS_266 genome is terminally redundant, and the studied phage uses a head-full strategy for DNA packaging (Supplementary Data S1). The AerS_266 genome sequence was deposited in the NCBI GenBank database (accession number OR496884).

**Figure 2:**
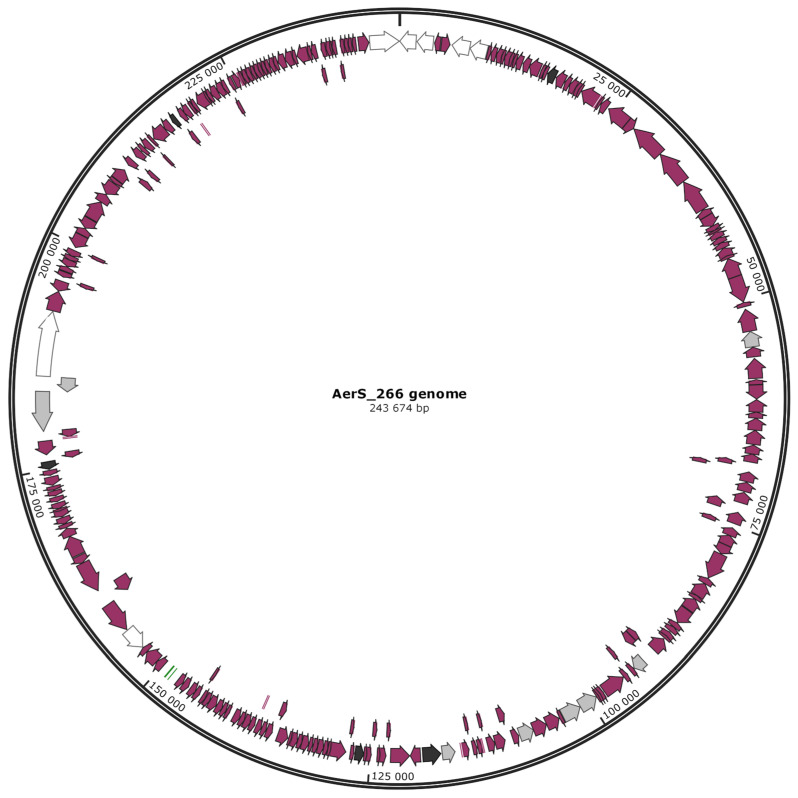
AerS_266 genome map; white arrows mark genes corresponding to RBPs (gp1, gp2, gp5, gp6, gp154, gp178, and gp250); light-gray arrows mark genes, corresponding to the subunits of both RNAPs (gp47, gp82, gp90, gp91, gp95, gp107, gp176, and gp177); black arrows mark genes corresponding to QueC, chimallin, PhuZ, head maturation protease, and FolA proteins (gp19, gp108, gp117, gp171, and gp206, respectively). All the genes noted above are listed in clockwise orientation from the beginning of the genome.

The AerS_266 genome contains 253 putative genes, and 3 of them correspond to tRNAs (Figure 2). Eighty-four genes encode proteins with predicted functions that were determined based on their amino acid sequences and domain structure similarity. The remaining 166 genes were defined as hypothetical. The phages that are supposed to be used for phage therapy should not transfer any virulence factors or antimicrobial resistance. So, the absence of these factors in the AerS_266 genome was verified using the VF and ARG databases, respectively. No genes encoding such proteins were identified. 

Putative multisubunit RNAPs (both virion RNA polymerase and nonvirion RNA polymerase) were identified, which is typical for phiKZ-like jumbo phages [20,34]. In addition, a set of genes encoding enzymatic proteins, namely SbcCD complex ATPase (gp 29), DnaB-like replicative helicase (gp 51), RNA helicase (gp 86), family B DNA polymerase (gp 110), and large subunit of terminase (gp 155), were revealed in the AerS_266 genome. These genes have been previously defined as the core genes specific to the phiKZ-like subgroup of jumbo phages [35]. 

Genes encoding chimallin and PhuZ proteins were detected, which provide a unique lifestyle of jumbo phages (Figure 2). Chimallin was previously found to be a major component of the nucleus-like compartment that protects replicating phage DNA from bacterial defense systems by serving as a physical barrier between phage DNA and bacterial enzymes [36]. Tubulin-like protein PhuZ forms microfilaments that place the “pseudonucleus” in the center of the cell and facilitate the transport of empty capsids from their assembly sites to the phage pseudonucleus to fill them with a newly synthesized DNA [36,37]. The AerS_266 genome contains seven genes encoding putative receptor binding proteins (RBPs), and six of these proteins include domains with glycosidase activity (Figure 2). This fact suggests that the phage AerS_266 has a structurally sophisticated adsorption complex, as it has been previously shown for some other giant phages [38]. 

PhiKZ-like phages are complex molecular machines, and a critical step in the morphogenesis of their capsids is the proteolysis of the proteins of the head, contributing to the conversion of the pro-head into a mature capsid. The AerS_266 genome encodes two putative proteases, one of which, gp171, is similar to phiKZ-like head maturation proteases that are highly conserved and responsible for the cleavage of multiple pro-head proteins [39]. Apparently, the AerS_266 capsids also undergo proteolytic maturation, similar to other phiKZ-like phages.

Some jumbo phages contain a complete or partial DNA modification system responsible for the synthesis of deazaguanine bases and their incorporation into DNA instead of guanine [19]. The genes that encode queuosine biosynthesis proteins QueC (gp 19) and FolA (gp 206) were found in the AerS_266 genome (Figure 2). In addition, the AerS_266 genome includes the gene corresponding to S-adenosyl-L-methionine-dependent methyltransferase (gp148), which is responsible for the DNA base methylation. The endonucleases *Apa*I (GGGCC^C), *Sal*I (G^TCGAC), and *Smi*I (ATTT^AAAT), whose recognition sites are found in the AerS_266 genome, were used to determine if the phage DNA is modified. Both *Sal*I and *Smi*I hydrolyzed the phage DNA, but *Apa*I did not (data available in Supplementary Figure S1). This suggests that the phage AerS_266 uses at least one DNA modification system.

### 3.3. Comparative Analysis of the AerS_266 Genome

**Figure 3:**
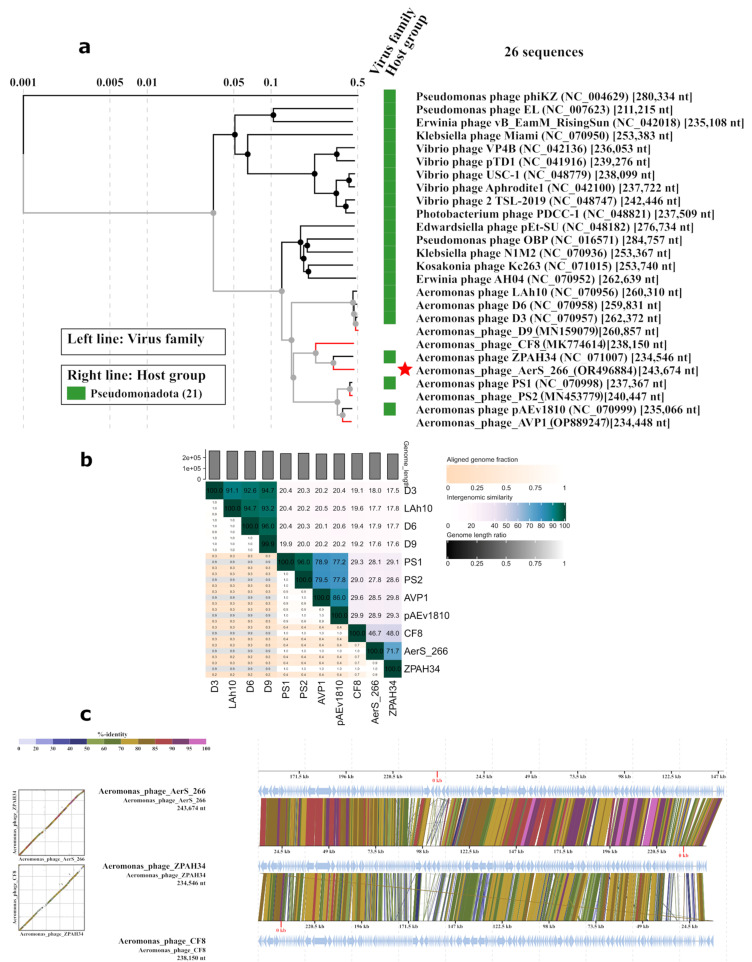
Comparative analysis of the AerS_266 genome with the most similar phage genomes: (**a**) The phylogenetic tree was obtained using the ViPTree tool; D9, CF8, PS2, and AVP1 genome sequences were extracted from NCBI GenBank and added to the analysis (marked with red lines). Phage AerS_266 is marked with an asterisk; (**b**) matrix of intergenomic similarities calculated using the VIRIDIC tool; (**c**) comparative genome alignment of the AerS_266, ZPAH34, and CF8 phages was obtained using the ViPTree tool.

Phylogenetic ViP tree analysis showed that the AerS_266 belongs to the clade of giant *Aeromonas* phages, whose genome lengths are more than 200 kb (Figure 3a). 

This clade contains D3, D6, D9, and LAh10 phages belonging to the *Ludhianavirus* genus; phages PS1, pAEv1810, PS2, and AVP1, grouped into the *Ferozepurvirus* genus; and ZPAH34 phage, which has been previously classified as the only member of the *Chaoshanvirus* genus [18]. In addition, one more phage, CF8 [40], was designated as a member of the order *Caudoviricetes*. All of them, with the exception of AVP1, were specific to *A. hydrophila* strains. The AVP1 phage had *A. veronii* as a host. AerS_266, the only phiKZ-like *A. salmonicida* phage in this clade, clustered with ZPAH34 and CF8 *A. hydrophila* phages in the phylogenetic tree (Figure 3a). The AerS_266 genome has 71.7% and 46.7% similarity to the genomes of the ZPAH34 and CF8 phages, respectively (Figure 3b); all three phages demonstrate high gene synteny (Figure 3c). At the same time, the level of similarity between ZPAH34 and AerS_266 genomes is close to the borderline separating two different genera [41]. Apparently, the taxonomic position of the phage AerS_266 would become clear when more jumbo phages of aeromonads are found.

**Figure 4:**
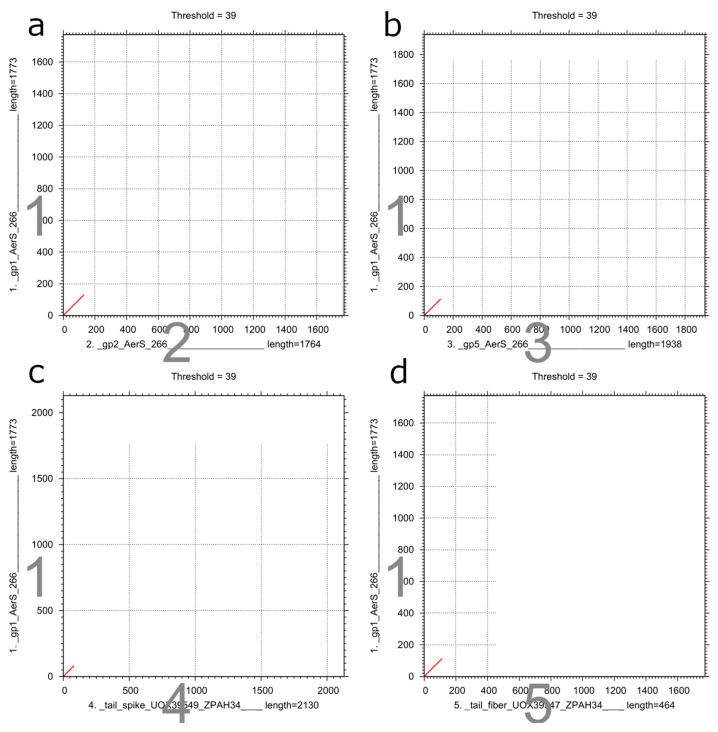
Dot-plot analysis of nucleotide sequences that encode RBPs of the AerS_266 and ZPAH34 phages. Analysis was performed using the MAFFT software. The names of gene products and the lengths of their corresponding genes are indicated along the axes of the plots. The red lines in the diagonal regions of the plots correspond to the location and length of similar fragments of genes: (**a**) comparison of genes, encoding gp1 and gp2 of the phage AerS_266; (**b**) comparison of genes, encoding gp1 and gp5 the phage AerS_266; (**c**) comparison of genes, encoding gp1 of the phage AerS_266 and tail fiber protein (UOX39547) of the phage ZPAH34; (**d**) comparison of genes, encoding gp1 of the phage AerS_266 and tail spike protein (UOX39549) of the phage ZPAH34.

Despite the high level of gene synteny between the AerS_266 and ZPAH34 genomes, a region of limited similarity was observed in the comparative alignment of their genomes (Figure 3c). According to the AerS_266 genomic map, it corresponded to the first 10 kb at the beginning of the genome (Figure 2 and Figure 3c). This region of the AerS_266 genome contained genes encoding putative tail spike proteins (gp1, gp2, gp5, and gp6). All these tail spike proteins showed various sequences; however, the dot-plot analysis revealed that gp1, gp2, and gp5 had a short similar fragment at the beginning of their genes (Figure 4a,b). In addition, BLASTX search against protein sequences deposited in NCBI GenBank demonstrated that the N-termini of gp1, gp2, and gp5 are similar to the N-termini of receptor binding proteins, RBPs (spike or fiber) of *Aeromonas* phage ZPAH34 (Figure 4c,d). Notably, gp6 showed no similarity with proteins of other *Aeromonas* phages.

The diversity of tail spike proteins with different receptor binding domains indicates that the phage AerS_266 is capable of binding several receptors on the bacterial surface. Apparently, the host spectra for the *Aeromonas* phages AerS_266 and ZPAH34 should be different. At the same time, the presence of similar N-termini between these proteins suggests that RBPs are attached to the conservative tail proteins of phages through these sequences.

### 3.4. Phylogenetic Analysis of the Essential Proteins of the Phage AerS_266

**Figure 5:**
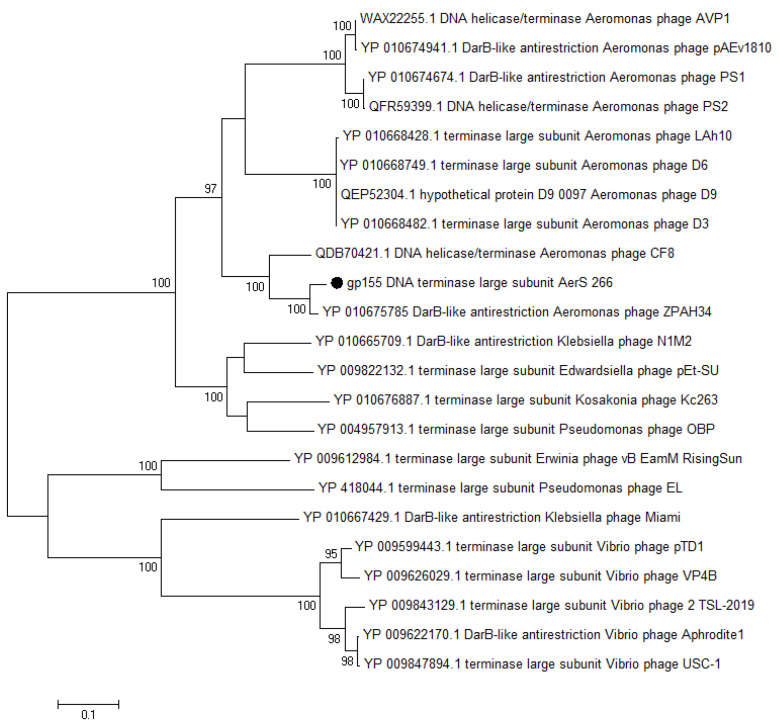
Phylogenetic analysis of the large subunit of terminase of the investigated phage AerS_266 with the most similar protein sequences. Alignment and analysis were performed using MEGA 11.0. The maximum likelihood method was used to construct the tree. Statistical support above 75% is shown at the nodes. The sequence ID of the large subunit of the AerS_266 terminase is marked with a black circle.

In order to confirm the taxonomy of the phage AerS_266, we performed a phylogenetic analysis of some essential phage proteins along with their most similar orthologs. The large subunit of terminase, capsid protein, and DNA polymerase were chosen for phylogenetic analysis. The obtained results corresponded to the results of a comparative genome analysis that was carried out using the ViPTree tool (Figure 3a). The phylogenetic trees constructed for all three proteins had similar topology; therefore, we present only one of them in the text (Figure 5), and two others are available in Supplementary Figure S2. As a result, the phylogenetic analysis of proteins confirms the data obtained using methods of comparative genomic analysis. 

In conclusion, a novel jumbo phage, AerS_266, infecting *A. salmonicida* was described for the first time. The phage AerS_266 is able to infect mesophilic strains of *A. salmonicida* species. The AerS_266 genome contains genomic determinants, characteristic of phiKZ-like phages, including multiple RNAP subunits, chimallin, and tubulin. The phage AerS_266 has a complex adsorption apparatus, and its DNA is modified. According to genomic comparative analysis, the AerS_266 genome is the most similar (71.7% similarity) to the genome of the *A. hydrophila* phage ZPAH34, which was previously classified as the only member of the genus *Chaoshanvirus*. 

The AerS_266 genome does not contain genes encoding undesirable virulence factors, antibiotic resistance, and phage integrases. This phage could thus potentially be used as an antimicrobial agent. The AerS_266 genome does not contain genes encoding undesirable virulence factors, antibiotic resistance, and phage integrases. This phage could thus potentially be used as an antimicrobial agent. To date, a phage cocktail, which consists of five *A. salmonicida* phages, namely AS-szw, AS-yj, AS-zj, AS-sw, and AS-gz (*Straboviridae* family), has been reported [14], and the *Aeromonas* phage ZPAH34 has already been used to prevent biofilm growth in vitro [18]. At the same time, it has been shown that giant phages are prone to mutations and rearrangements in the genome, and some of them are transducing phages and can exist in pseudolysogenic stage in bacterial cells [42,43,44,45]. Notably, the phage AerS_266 has a slow lytic life cycle and a relatively narrow host range. Therefore, the effectiveness and safety of its application as an antimicrobial agent should be further investigated. 

## Data Availability

Not applicable.

## Supplementary Materials

The following supporting information can be downloaded at: https://www.mdpi.com/article/10.3390/microorganisms11112649/s1, Figure S1: Agarose gel electrophoresis of the phage AerS_266 DNA hydrolysis; Figure S2: Phylogenetic analysis of essential phage proteins: (a) capsid protein, (b) DNA polymerase; Table S1: *Aeromonas* spp. strains screened in host range assay; Data S1: Phage AerS_266 genome termini identification using PhageTerm tool; Data S2: The AerS_266 genome annotation.
