# Supplementary material for: Isolation, Characterization and Genomic Analysis of a Novel Jumbo Phage, AerS_266, That Infects Aeromonas salmonicida
Source: Microorganisms. 2023 Oct 28;11(11):2649. doi: 10.3390/microorganisms11112649 (PMC10673249; doi:10.3390/microorganisms11112649)
Supplement: Supplementary file 1 [file microorganisms-11-02649-s001.zip › Figure S1.pdf]

Figure S1. Agarose gel-electrophoresis of the phage AerS_266 DNA hydrolysis

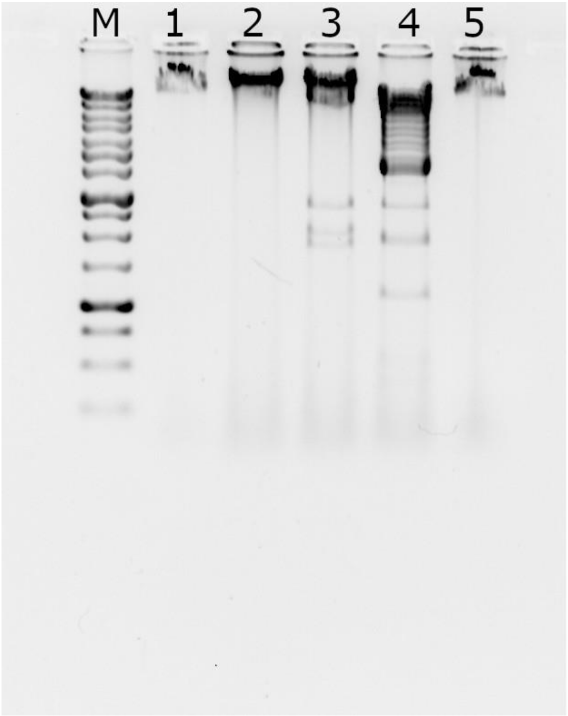

Lines: M – DNA ladder 50kb (SibEnzyme, Russia); 1, 5 – intact AerS_266 DNA; 2 – DNA hydrolysis using *Apa*I; 3 - DNA hydrolysis using *Sal*I; 4 - DNA hydrolysis using *Smi*I
