# Supplementary material for: Isolation, Characterization and Genomic Analysis of a Novel Jumbo Phage, AerS_266, That Infects Aeromonas salmonicida
Source: Microorganisms. 2023 Oct 28;11(11):2649. doi: 10.3390/microorganisms11112649 (PMC10673249; doi:10.3390/microorganisms11112649)
Supplement: Supplementary file 1 [file microorganisms-11-02649-s001.zip › Figure S2.pdf]

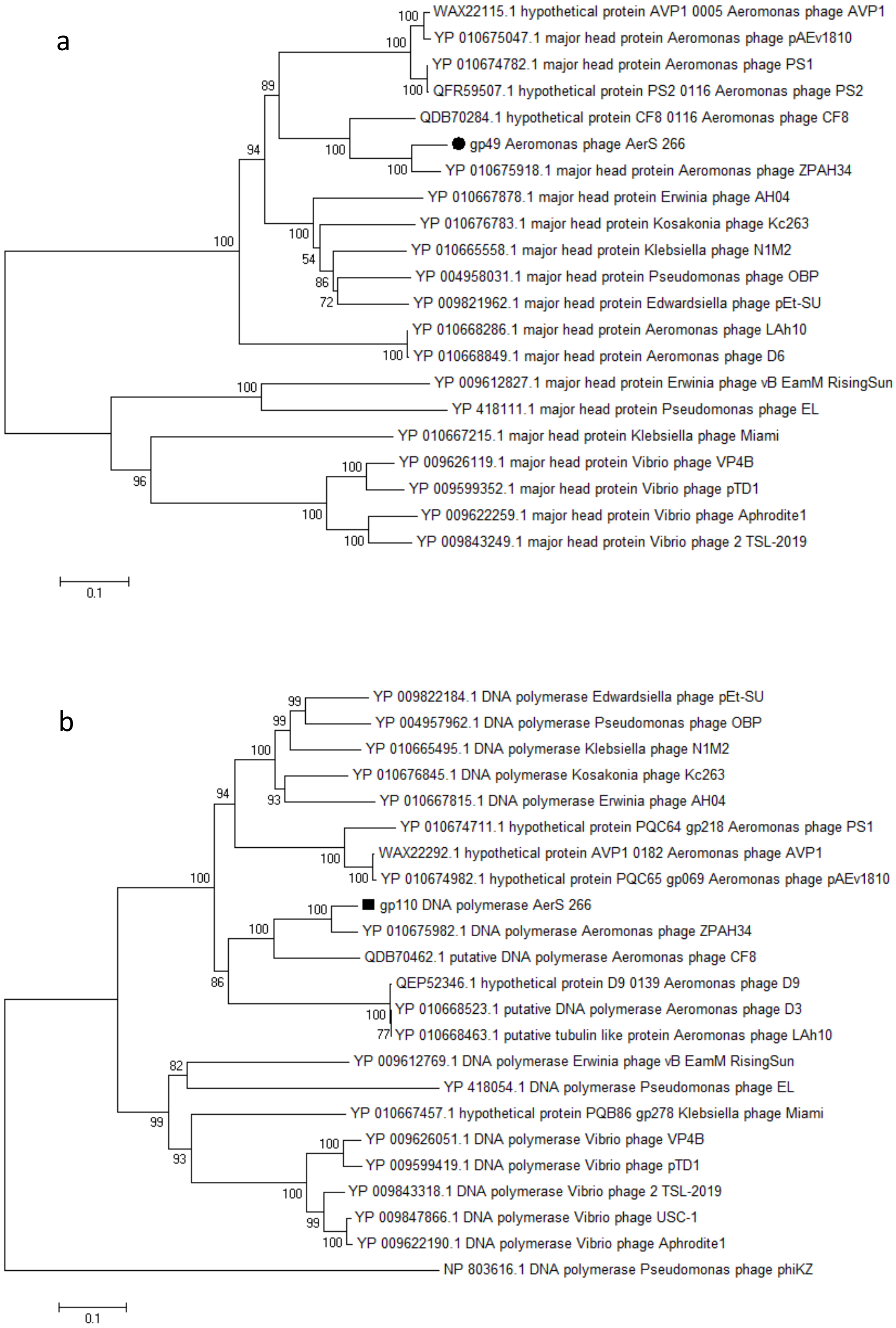

Figure S2. Phylogenetic analysis of the proteins of the phage AerS_266 with the most similar protein sequences. Alignment and analysis were performed using MEGA 11.0. The Maximum Likelihood method was used to construct the tree. (a) capsid protein, the sequence ID of the capsid protein of the AerS_266 phage is marked with black circle; (b) DNA polymerase; the sequence ID of DNA polymerase of the AerS_266 phage is marked with black box
