# Supplementary material for: Isolation, Characterization and Genomic Analysis of a Novel Jumbo Phage, AerS_266, That Infects Aeromonas salmonicida
Source: Microorganisms. 2023 Oct 28;11(11):2649. doi: 10.3390/microorganisms11112649 (PMC10673249; doi:10.3390/microorganisms11112649)
Supplement: Supplementary file 1 [file microorganisms-11-02649-s001.zip › Table S1.pdf]

Table S1. *Aeromonas* spp. strains screened in host range assay

No	Species_16S	CEMTC No	Isolation source	Temperature of growth, ºC	NCBI GenBank ID for 16S rRNA
1	*A. allosaccharophila*	7413	Lake in Novosibirsk region, Siberia, Russia	25	OQ332360
2	*A. caviae*	2395	Waste waters in Phuket, Thailand	37	OP218045
3	*A. hydrophila*	2094	River in Dalat, Vietnam	30	OP849657
4	*A. hydrophila*	6279	Lake in Kemerovo region, Siberia, Russia	25	OQ332358
5	*A. hydrophila*	7426	Pond in Primorsky region, Far-East of Russia	25	OQ332361
6	*A. hydrophila*	8139	Pond in Adler, Krasnodar region, Russia	25	OQ834572
7	*A. jandaei*	1458	Waterfall in North Goa, India	37	ON479599
8	*A. media*	5475	River in Novosibirsk region, Siberia, Russia	25	OP659002
9	*A. media*	7654	River in Novosibirsk region, Siberia, Russia	25	OQ332364
10	*A. media*	8112	Pond in Adler, Krasnodar region, Russia	25	OQ834573
11	*A. media*	8147	River in Omsk region, Siberia, Russia	25	OQ913697
12	*A. popoffii*	1430	River in the Death Valley, Kamchatka peninsula, Russia	30	ON479598
13	*A. popoffii*	3381	Lake in Ulagan region, Altay republic, Siberia, Russia	22	ON479601
14	*A. popoffii*	4062	River in Novosibirsk region, Siberia, Russia	28	ON479602
15	*A. rivipollensis*	6434	Lake in Kazan, Tatarstan republic, Russia	25	OP659005
16	*A. salmonicida*	1370	Lake in Altay region, Siberia, Russia	37	OP849654
17	*A. salmonicida*	3364	Lake in Ulagan region, Altay republic, Siberia, Russia	37	ON479600
18	***A. salmonicida***	**3375***	Lake in Ulagan region, Altay republic, Siberia, Russia	25	OR226260
19	*A. salmonicida*	4331	Lake in Novosibirsk region, Siberia, Russia	25	OP849658
20	***A. salmonicida***	**4537****	Pond in Novosibirsk region, Siberia, Russia	25	OP218048
21	*A. salmonicida*	4546	Mineral water spring, Sakhalin region, Far-East of Russia	25	ON479605
22	*A. salmonicida*	5456	Lake in Ulagan region, Altay republic, Siberia, Russia	22	OP218051
23	***A. salmonicida***	**5599**	Pond in Novosibirsk region, Siberia, Russia	25	OP849659
24	*A. salmonicida*	5828	Swamp in Altay region, Siberia, Russia	25	OP849660
25	*A. salmonicida*	5875	River in Altay region, Siberia, Russia	25	OR226261
26	*A. salmonicida*	7512	Swamp in Novosibirsk region, Siberia, Russia	25	OQ332363
27	***A. salmonicida***	**7722**	River in Novosibirsk region, Siberia, Russia	25	OQ913695
28	*A. salmonicida*	8183	Coastal area of the river, Omsk region, Siberia, Russia	25	OQ913698
29	*Aeromonas sp.*	1369	Lake in Altay region, Siberia, Russia	37	OP218042
30	*Aeromonas sp.*	1354	Lake in Altay region, Siberia, Russia	37	OP849653
31	*Aeromonas sp.*	4529	Pond in Novosibirsk region, Siberia, Russia	25	OP218047
32	*A. veronii*	1362	Lake in Altay region, Siberia, Russia	37	ON479597

33	*A. veronii*	1414	Thermal field in Caldera Uzon, Kamchatka peninsula, Far-East of Russia	30	OP849655
34	*A. veronii*	1416	Thermal field in Caldera Uzon, Kamchatka peninsula, Far-East of Russia	30	OP218043
35	*A. veronii*	5502	River in Ongudai region, Altay republic, Siberia, Russia	25	OQ332355
36	*A. veronii*	6243	River in Kazan, Tatarstan republic, Russia	25	OQ332357
37	*A. veronii*	6289	Spring in Kemerovo region, Siberia, Russia	25	OQ332359
38	*A. veronii*	4064	River in Novosibirsk region, Siberia, Russia	28	ON479603
39	*A. veronii*	6436	Lake in Kazan, Tatarstan republic, Russia	25	OP659006
40	*A. veronii*	6618	Pond in Novosibirsk region, Russia	25	OP659007
41	*A. veronii*	7112	River in Kazan, Tatarstan republic, Russia	25	OQ913692
42	*A. veronii*	7445	Pond in Adler, Krasnodar region, Russia	25	OQ332362
43	*A. veronii*	7500	Swamp in Novosibirsk region, Siberia, Russia	25	OQ913693
44	*A. veronii*	7638	Lake in Novosibirsk region, Siberia, Russia	25	OQ913694
45	*A. veronii*	8132	Pond in Adler, Krasnodar region, Russia	25	OQ913696

*Susceptible strains are marked with bold, **Host strain for phage AerS_266
